# Correlation Between Vitamin E Levels and Cholesterol, Vitamin D, and Frequency of Pulmonary Exacerbations in Children With Cystic Fibrosis

**DOI:** 10.7759/cureus.73562

**Published:** 2024-11-12

**Authors:** Hasan M Isa, Fatema A Alkharsi, Zahra S Mohamed, Zahra H Isa, Batool H Isa, Mahmood J Ali, Afaf M Mohamed

**Affiliations:** 1 Department of Pediatrics, Arabian Gulf University, Manama, BHR; 2 Department of Pediatrics, Salmaniya Medical Complex, Manama, BHR; 3 Department of Pediatrics, Royal College of Surgeons in Ireland, Manama, BHR; 4 Department of Public Health, The Ministry of Health, Manama, BHR

**Keywords:** bahrain, cholesterol level, cystic fibrosis, pulmonary exacerbation, vitamin d deficiency, vitamin e deficiency

## Abstract

Introduction

Children with cystic fibrosis (CF) have lipid maldigestion due to pancreatic insufficiency, which causes malabsorption of fat-soluble vitamins. The primary objective of this study was to assess the prevalence of vitamin E deficiency among children with CF. The secondary objective was to examine the correlation between vitamin E levels with demographic data, laboratory findings, and the number of pulmonary exacerbations. Furthermore, the study aimed to identify potential predictors of vitamin E deficiency in this population.

Methods

A prospective cohort study was conducted from July 1, 2017, to April 30, 2019. Medical records of children diagnosed with CF at the Department of Pediatrics, Salmaniya Medical Complex, Bahrain were reviewed. Patients who didn’t receive fat-soluble vitamin supplementation for at least three days were recruited for the study. Light-protected blood samples were tested for vitamin E and D levels and fasting serum cholesterol levels. Patients with vitamin E deficiency were compared with those without regarding demography, laboratory results, and number of pulmonary exacerbations.

Results

Of 109 patients with CF, 35 (32.1%) fulfilled the inclusion criteria. Eighteen (51.4%) were males. The mean age was 6.8 ± 4.7 years. Eleven (31.4%) patients were symptomatic. Vitamin E and D were deficient in nine (25.7%) and 28/34 (82.4%) patients, respectively. Cholesterol was low in 29 (82.9%). The mean vitamin E level in the deficient group was significantly lower (3.2 ± 1.7 mg/L) than that (10.3 ± 3.1 mg/L) of the vitamin E-sufficient group (P < 0.0001). A significant negative correlation was noted between vitamin E levels and white blood cell (WBC) count (r = -0.408; P = 0.015). However, no correlation was found between vitamin E levels and cholesterol, vitamin D levels, or the number of pulmonary exacerbations. Vitamin E-deficient patients had lower weight at presentation (P = 0.045), hemoglobin level (P = 0.001), and salbutamol use (P = 0.022), but higher reticulocyte percentage (P = 0.034) and WBC count (P = 0.001) compared to the vitamin E-sufficient group.

Conclusion

Vitamin E deficiency is common among patients with CF in Bahrain and may increase the risk of hemolytic anemia. This deficiency did not seem to affect the frequency of pulmonary exacerbations. Management of vitamin E deficiency in patients with CF should be hastened to avoid irreversible complications.

## Introduction

Cystic fibrosis (CF) is an autosomal recessive, multi-system, life-threatening disorder [1,2]. As a result of exocrine pancreatic insufficiency, approximately 85% of patients with CF show evidence of maldigestion of lipids and proteins, which subsequently causes malabsorption of fat-soluble vitamins, including vitamin E [3,4]. Patients with CF have an impaired balance between prooxidants and antioxidants due to chronic infection and inflammatory processes, which increase the free radical loads [1]. Alpha-tocopherol, a form of vitamin E, is one of the most potent free radical scavengers in the human body, and it is absorbed from the gastrointestinal tract as a fat-soluble vitamin [5,6]. Adequate vitamin E levels are essential for the body to function normally [5,6]. The role of vitamin E involves the prevention of cell membrane oxidation and maintaining neurological functions, and it also has a role in the cognitive functions of infants [7,8].

Vitamin E deficiency is associated with progressive hyporeflexia or areflexia, ataxia, peripheral neuropathy, loss of vision, and hemolytic anemia [5]. Moreover, reduced serum levels of vitamin E are linked to a higher risk of pulmonary exacerbations in patients with CF, even when these levels are at the lower limit of the normal range [9]. However, no clear evidence was found that higher vitamin E levels had protective effects on pulmonary functions [10]. Nonetheless, in the Middle East, a notable retrospective prospective study was conducted in 2007 to investigate the long-term effects of serum vitamin A and E levels on the incidence of pulmonary exacerbations among patients with CF [9]. This study involved 102 participants, of whom 74 (72.5%) were identified as having pancreatic insufficiency. Throughout the study, a total of 597 pulmonary exacerbations were recorded [9]. The results indicated a correlation between lower serum levels of vitamins A and E and an increased frequency of pulmonary exacerbations [9].

Measuring vitamin E levels in the blood is necessary to diagnose its deficiency and to guide its management [5]. Treatment of vitamin E deficiency in children with CF can be achieved by fat-soluble vitamin supplementation in a water-soluble form, either separate or in combination with other vitamins [11]. Vitamin E can be given via oral, intravenous, and intramuscular routes and as a supplement in total parenteral nutrition [12]. Oral vitamin E seems safe and can restore or maintain sufficient serum levels in most children [13]. Therefore, painful intramuscular vitamin E injections can be avoided in these children [14]. Synthetic water-soluble form of vitamin E showed better bioavailability than water-miscible vitamin E in children with CF [12,15].

In Arab countries, most studies about CF focused on the incidence, clinical presentation, and types of mutations associated with CF [16]. This also applies to Bahrain, where four studies were published about CF and none specifically focused on vitamin E in this group of patients [17-20]. Accordingly, the primary objective of this study was to assess the prevalence of vitamin E deficiency among children with CF. The secondary objective was to examine the correlation between vitamin E levels with demographic data, laboratory findings, and the number of pulmonary exacerbations. Furthermore, the study aimed to identify potential predictors of vitamin E deficiency in this population. The present study is essential for addressing the existing knowledge gap concerning the prevalence of vitamin E deficiency among patients with CF in this geographical area, as such information is not thoroughly documented. Moreover, the outcomes of this research can provide valuable guidance for healthcare providers in identifying and managing patients who are at risk, thereby enhancing patient care.

## Materials and methods

Study design and setting

In this prospective cohort study, we reviewed medical records of all pediatric patients with CF who attended the CF multidisciplinary clinics (twice per month) or were admitted to the general pediatric wards at the Department of Pediatrics, Salmaniya Medical Complex, Kingdom of Bahrain between July 1, 2017 and April 30, 2019. Our hospital is recognized as the leading tertiary care center in Bahrain, where all patients with CF requiring multidisciplinary care are referred for diagnosis and management. Patients with CF receive comprehensive, multidisciplinary care from a team that includes pediatric gastroenterologists, pediatric pulmonologists, physiotherapists, and specialized pediatric dietitians, all of whom conduct evaluations on the same day every three months. Moreover, other subspecialties, such as infectious disease specialists, endocrinologists, social workers, and others, are also involved when needed. To confirm the diagnosis, sweat chloride test and genetic testing are performed for all suspected cases of CF.

Study population

**Figure 1:**
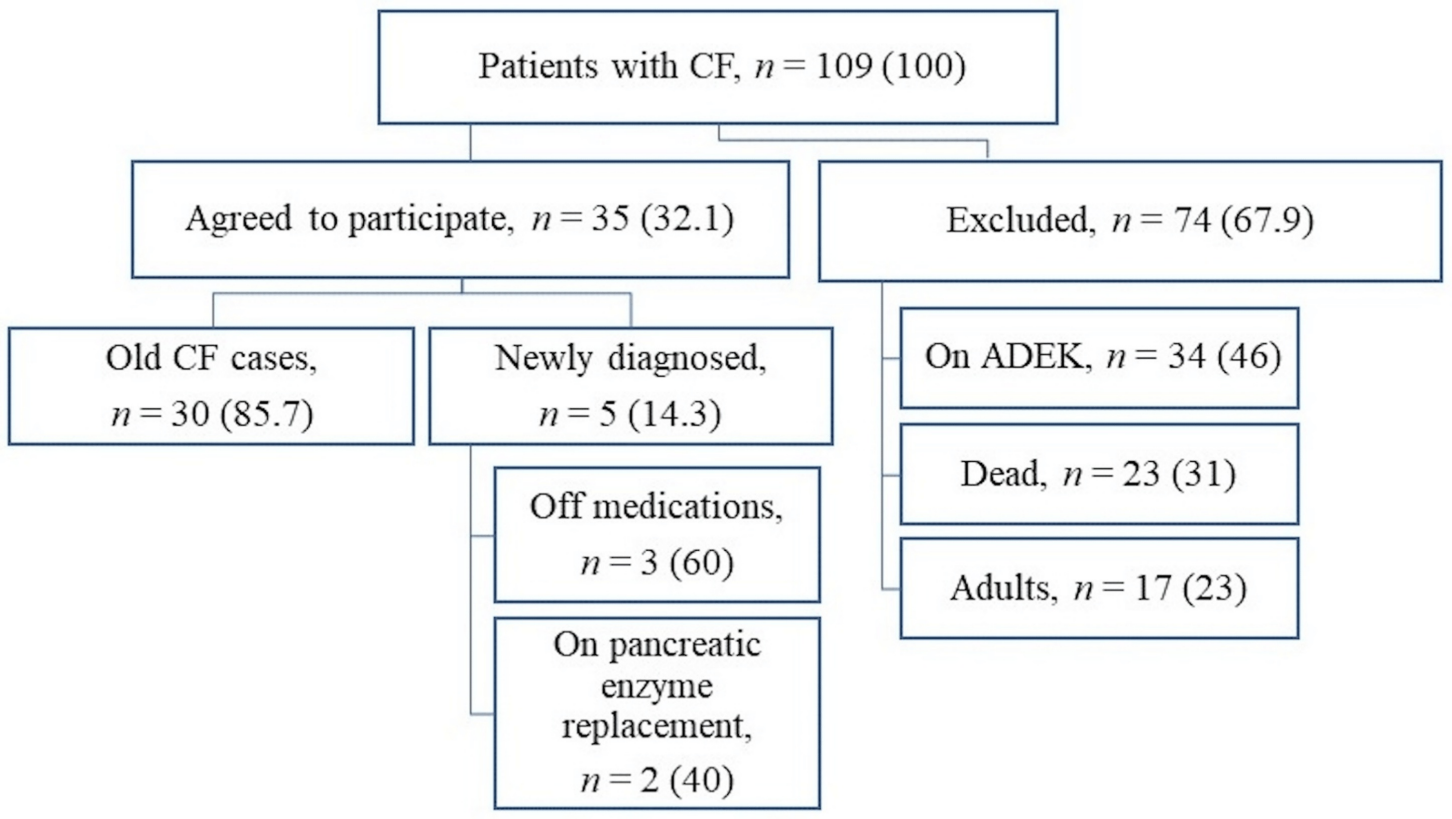
Flowchart illustrating the recruitment process of pediatric patients with cystic fibrosis Image credits: Hasan M. Isa, Zahraa S. Mohamed Data are presented as numbers (%). CF: cystic fibrosis; ADEK: fat-soluble vitamins A, D, E, and K supplementation

This study included only newly diagnosed patients before starting any vitamin supplementation during the study period, and the old patients with CF who did not receive any fat-soluble vitamin supplementation for at least three days before the time of the study due to either poor compliance or unavailability of these vitamins. Diagnosis of CF was made based on the standard criteria (clinical findings consistent with CF, two positive sweat chloride tests by pilocarpine iontophoresis more than 60 mEq/L, and/or positive CF transmembrane conductance regulator (CFTR) gene mutations analysis) [21]. Patients were excluded from the study if they had received fat-soluble vitamins within the last three days before the time of the study, dead patients, those above 18 years of age, and those who had other gastrointestinal diseases or any medical condition that can affect the absorption of these vitamins, such as other pancreatic diseases, chronic cholestasis, celiac disease, abetalipoproteinemia, intestinal lymphangiectasia, short bowel disorder, and inherited defects in bile acid synthesis [22]. All patients with CF who fulfilled the study inclusion criteria were recruited. The recruitment process is detailed in Figure 1.

Data collection

All 35 patients agreed to participate in the study. Patients who were already admitted to the hospital were interviewed and examined. Those at home were invited to the Department of Pediatrics for an interview regarding past medical history, having new symptoms, and to undergo a physical examination. Demographic data were collected through direct interviews with the patient’s parents/guardians and by reviewing the electronic medical records. The same senior pediatric gastroenterologist conducted patient interviews and performed thorough physical examinations, using a detailed history and physical examination checklist to ensure standardized assessment. Data included patients’ sex, age at presentation, age at the time of study, nationality, clinical presentation, growth parameters (weight, height, and body mass index (BMI) (kg/m^2^; where patient's weight is represented in kilograms and their height is in meters squared)), medications used, and last date patient was on vitamin E supplementations before the enrollment day, number of previous pulmonary exacerbations, number of previous hospitalizations, and intensive care unit admissions.

Laboratory testing

All the patients had the basic laboratory tests performed, including complete blood count, reticulocyte percentage, liver function tests (LFT), and vitamin D levels. A vitamin D level of 50 nmol/L or more was considered normal. An arrangement was also made with professional personnel from an outside laboratory (Alborg Medical Laboratories, Manama, Bahrain) before blood collection as vitamin E level testing is not routinely performed in our hospital. Blood samples were collected for both vitamin E and fasting serum cholesterol levels and placed in two ethylenediaminetetraacetic acid (EDTA) purple bottles to provide at least 2-4 ml of blood serum each. The blood samples were covered by aluminum sheets to be protected from direct sunlight, then kept in a special container and sent by the laboratory driver immediately to the laboratory for testing. Vitamin E level was determined by measuring alpha-tocopherol serum levels using the high-performance liquid chromatography method. The unit used to describe vitamin E level was mg/L, with the normal range of 2.9-16.6 mg/L for children two years and below and 5.7-19.9 mg/L for older children. Quest Diagnostics Nichols Institute, Valencia, developed this test and identified its analytical performance characteristics. This assay has been validated according to the Clinical Laboratory Improvement Amendments (CLIA) regulations and is used for clinical purposes. Fasting serum cholesterol levels were measured in mg/dL, with a normal range of 139-200 mg/dL. The laboratory results were available within two weeks.

Statistical analysis

The patient data were analyzed using IBM SPSS Statistics for Windows, Version 28 (Released 2021; IBM Corp., Armonk, New York, United States). The frequencies and percentages were calculated for categorical variables. Continuous variables were presented as mean and standard deviation (SD) or median and interquartile range (IQR) according to their distribution normality. Patients were divided into two groups based on the presence or absence of vitamin E deficiency. Both groups were compared in terms of demographic data and laboratory test results. Fisher's exact test or Pearson's chi-square test was used to compare categorical variables. A Mann-Whitney U test or student t-test was used to compare continuous variables. Vitamin E levels were correlated with the fasting cholesterol levels, vitamin D levels, white blood cell (WBC) count, hemoglobin level, reticulocyte percentage, number of hospital admissions, and number of pulmonary exacerbations using Spearman’s correlation coefficient (r_s_). The coefficient of determination (r^2^) and the simple regression equation were calculated. Variables found to be significant in univariate analysis and have no multicollinearity using a variation inflation factor were included in a binary logistic regression to identify the independent risk factors of vitamin E deficiency. Odds ratios (ORs) were used to assess the association between vitamin E status and other variables. The confidence level was set at 95% with a 5% confidence interval (CI). The P-value was considered statistically significant if <0.05.

Ethical clearance

This study was conducted according to the principles outlined in the Helsinki Declaration of 1995 (as revised in Edinburgh 2000). Ethical approval was granted by the Research Committee for Government Hospitals (IRB number: 56230522) at Salmaniya Medical Complex, Kingdom of Bahrain. Informed consent was obtained from the patient’s parents/guardians before enrollment in the study, and their confidentiality was maintained. The blood test results have been informed to the patient’s parents/guardians, and they were counseled accordingly.

## Results

During the study period, a total of 109 patients with CF were reviewed; of them, 35 (32.1%) patients were included in the analysis, of whom 18 (51.4%) were identified as males. Bahraini patients contributed the highest portion of the patients (n = 29, 82.9%), while six (17.1%) patients were non-Bahraini (two were from Jordan and one each from India, Syria, Pakistan, and Yemen). The mean age at the time of the study was 6.8 ± 4.7 years. Most of the patients were below the age of 10 years (n = 26, 74.2%). The median number of previous admissions was three (IQR: 2-7). Three (8.6%) patients had never been admitted before, as they were diagnosed as outpatients based on positive family history. Vitamin E was deficient in nine (25.7%) patients, while 26 (74.3%) had normal levels (Table 1).

**Table 1:** Demographic data of patients with cystic fibrosis and vitamin E deficiency and those with normal levels Data are presented as number (%), mean ± standard deviation, or median (interquartile range). ^a^Fisher’s exact test; ^b^Mann-Whitney U test; ^c^Student t-test; ^d^Pearson's chi-square P-value < 0.05 is considered statistically significant. Bold values indicate statistical significance. IQR: interquartile range; SD: standard deviation; BMI: body mass index; ADEK: fat-soluble vitamins A, D, E, and K

Variables	Vitamin E Level		P-value	Total, n = 35 (100)
	Low, n = 9 (25.7)	Normal, n = 26 (74.3)		
Sex Male	3 (33.3)	15 (57.7)	0.264^a^	18 (51.4)
Female	6 (66.7)	11 (42.3)		17 (48.6)
Age at presentation (y), median (IQR)	0.4 (0.0-10.3)	0.3 (0.2-0.5)	0.763^b^	0.3 (0.2-0.5)
Age at time of study (y), mean ± SD	4.5 ± 5.5	7.6 ± 4.2	0.09^c^ (CI: -6.7, 0.5)	6.8 ± 4.7
Age groups (y) 0-4.9	6 (66.7)	7 (26.9)	0.145^d^	13 (37.1)
5-9.9	1 (11.1)	12 (46.2)		13 (37.1)
10-14.9	2 (22.2)	6 (23.1)		8 (22.9)
15-18	0 (0.0)	1 (3.8)		1 (2.9)
Nationality Bahraini	8 (88.9)	21 (80.8)	1.000^a^	29 (82.9)
Non-Bahraini	1 (11.1)	5 (19.2)		6 (17.1)
Height at presentation (cm), median (IQR)	76 (67-106)	125 (94-131)	0.681^b^	117 (98-131)
Weight at presentation (kg), mean ± SD	12.7 ± 9.0	20.2 ± 9.4	0.045^c^ (CI: 0.18, 14.8)	18.2 ± 9.7
BMI (kg/m^2^), mean ± SD	14.4 ± 2.4	14.2 ± 1.9	0.793^c^ (CI: -1.9, 1.3)	14.3 ± 1.9
Off ADEK duration (mo), median (IQR)	2 (0.5-24)	5.5 (2-12)	0.786^b^	3 (0.9-12)
Previous gastrointestinal surgery	2 (22.2)	5 (19.2)	1.000^a^	7 (20)
Number of hospital admissions, median (IQR)	4 (3-6)	3 (2-7)	0.323^b^	3 (2-7)
Number of pulmonary exacerbations, median (IQR)	4 (2-6)	3 (1-6)	0.305^b^	3 (1-6)

Considering demographic data, no significant differences were found between the two groups apart from lower weight at presentation in the vitamin E-deficient patients (P = 0.045). Also, the number of previous admissions and pulmonary exacerbations did not differ between the two study groups.

The clinical symptoms and signs at the initial disease presentation are shown in Table 2.

**Table 2:** Initial clinical presentations of children with cystic fibrosis Data are presented as numbers (%). ^a^Fisher’s exact test P-value < 0.05 is considered statistically significant. ^*^Some patients had more than one clinical presentation.

Clinical presentation	Vitamin E Level		P-value^a^	Total, n = 35 (100)^*^
	Low, n = 9 (25.7)	Normal, n = 26 (74.3)		
Cough	7 (77.8)	22 (84.6)	0.635	29 (82.9)
Weight faltering	6 (85.7)	21 (80.8)	0.396	27 (77.1)
Steatorrhea	4 (44.4)	13 (50)	1.000	17 (48.6)
Anorexia	4 (44.4)	11 (42.3)	1.000	15 (42.9)
Clubbing	2 (22.2)	9 (34.6)	0.685	11 (31.4)
Constipation	1 (11.1)	7 (26.9)	0.648	8 (22.9)
Distal intestinal obstruction syndrome	2 (22.2)	2 (7.8)	0.268	4 (15.4)
Chronic rhinitis	0 (0.0)	4 (15.4)	0.553	4 (15.4)
Rectal prolapse	1 (11.1)	1 (3.9)	0.454	2 (5.7)
Dysphagia	0 (0.0)	1 (3.9)	1.000	1 (2.9)

Most of the patients presented with cough, weight faltering, and steatorrhea. There were no significant differences in clinical presentations between the two groups. At the time of blood collection, 24 (68.6%) patients were asymptomatic, while 11 (31.4%) had one or two symptoms. Four patients (11.2%) had loose motions/steatorrhea, three (8.6%) had active cough, two (5.7%) had slow weight gain, one (2.9%) had poor appetite and occasional cough, and one (2.9%) had upper respiratory tract infection. On physical examination, 26 (74.3%) patients had no abnormal findings, and nine (25.7%) had one or more positive findings, including clubbing in eight (22.9%), abdominal distension, cyanosis, café au lait spot, and left nasal polyps each in one patient (2.9%). None of the patients displayed clinical signs of neuropathy at the time of the study.

Regarding associated diseases, two patients (5.7%) had a previous history of cow's milk protein allergy; however, they were asymptomatic at the time of the study. The other two patients (5.7%) developed Methicillin-Resistant *Staphylococcus aureus* pneumonia. *Helicobacter pylori* gastritis, subclinical hypothyroidism, delayed speech, and nocturnal enuresis were found in one patient (2.9%). Seven patients (20%) underwent previous surgical interventions (laparotomy for distal intestinal obstruction syndrome (n = 4, 11.4%), while appendectomy, inguinal hernia repair, and nasal polyps’ removal (n = 1, 2.9%, each)).

Medications prescribed for the patients after the diagnosis are shown in Table 3.

**Table 3:** Medications used in patients with cystic fibrosis Data are presented as numbers (%). ^a^Fisher’s exact test P-value < 0.05 is considered statistically significant. Bold values indicate statistical significance. *These vitamins were stopped at least three days before enrollment in the study. ADEK: fat-soluble vitamins A, D, E, and K

Medication used	Vitamin E Level		P-value^a^	Total, n = 35 (100)
	Low, n = 9 (25.7)	Normal, n = 26 (74.3)		
Pancreatic enzyme replacement therapy	8 (88.9)	24 (92.3)	1.000	32 (91.4)
Previous ADEK supplementation^*^	6 (66.7)	24 (92.3)	0.095	30 (85.7)
Bronchodilator (salbutamol)	1 (11.1)	15 (57.7)	0.022	16 (45.7)
Dornase alfa	4 (44.4)	10 (38.5)	1.000	14 (40.0)
Oral antibiotic (azithromycin)	4 (44.4)	8 (30.8)	0.685	12 (34.3)
Multivitamins	2 (22.2)	8 (30.8)	1.000	10 (28.6)
Inhaled antibiotic (tobramycin)	2 (22.2)	8 (30.8)	1.000	10 (28.6)
Proton pump inhibitor	2 (22.2)	3 (11.5)	0.586	5 (14.3)
Inhaled steroid	0 (0.0)	5 (19.2)	0.297	5 (14.3)
Ursodeoxycholic acid	2 (22.2)	2 (7.7)	0.268	4 (11.4)
Vitamin D supplementation	2 (22.2)	2 (7.7)	0.268	4 (11.4)
Iron supplementation	0 (0.0)	1 (3.8)	1.000	1 (2.9)

All the patients were off fat-soluble vitamins for at least three days before the time of the study. The median duration since the last fat-soluble vitamin intake was three (IQR: 0.9-12) months. Of the 35 patients, 30 (85.7%) were previously treated, and five (14.3%) were newly diagnosed at the time of the study. The previously treated patients received pancreatic enzyme replacement therapy (Creon) and fat-soluble vitamins A, D, E, and K (ADEK) supplementation but stopped taking ADEK supplementation due to unavailability of the medications (n = 27, 77.1%) or poor compliance (n = 3, 8.5%). The new patients were not started on ADEK supplementation yet, but two of them received pancreatic enzyme replacement therapy. There were no significant differences in the medical therapy between the two vitamin E groups apart from lower salbutamol use in the vitamin E-deficient patients (P = 0.022).

**Figure 2:**
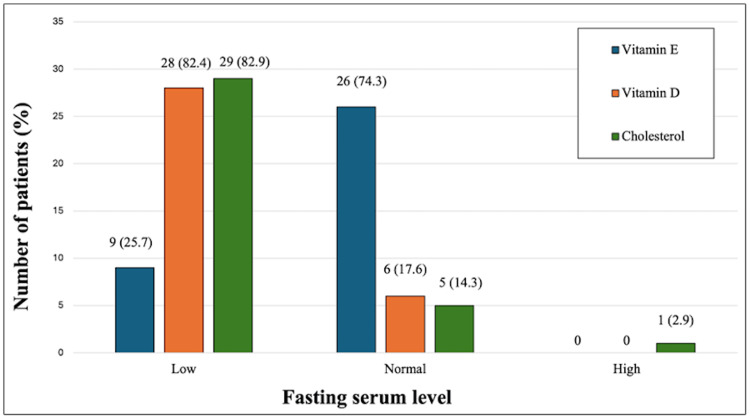
Fasting serum levels of vitamin E, vitamin D, and cholesterol in children with cystic fibrosis Image credits: Hasan M. Isa, Fatema A. Alkharsi, Zahraa S. Mohamed, Zahra H. Isa, Batool H. Isa, Mahmood J. Ali, Afaf M. Mohamed

Results of fasting serum levels of vitamin E, vitamin D, and cholesterol are shown in Figure 2.

The overall mean vitamin E level was 8.5 ± 4.2 mg/L. All five newly diagnosed patients were vitamin E-deficient. Three (8.6%) patients had severe vitamin E deficiency with levels below 2 mg/L (two had a level of 1 mg/L, and one had a 1.7 mg/L level). The mean vitamin E level in the deficient group was 3.2 ± 1.7 mg/L compared to 10.3 ± 3.1 mg/L in the vitamin E-sufficient group (P < 0.0001, CI: -9.3, -4.9).

Vitamin D was deficient in 28/34 (82.4%) patients. The overall mean vitamin D level was 37.7 ± 16.4 nmol/L. There was no significant difference between vitamin E-deficient patients and those with normal levels in terms of vitamin D deficiency (8/9 (88.9%) versus 20/25 (80%), respectively, P = 1.000). All four patients who received prophylactic vitamin D supplementation (400 IU daily) were vitamin D-deficient, with a level of 21, 28, 41, and 41 nmol/L.

Serum cholesterol level was low in 29 (82.9%) patients. The overall mean serum cholesterol level was 117.8 ± 27.8 mg/dL. There was no significant difference between vitamin E-deficient patients and those with normal levels in terms of low cholesterol levels (8/9 (88.9%) versus 21/26 (80.8%), respectively, P = 0.785).

**Figure 3:**
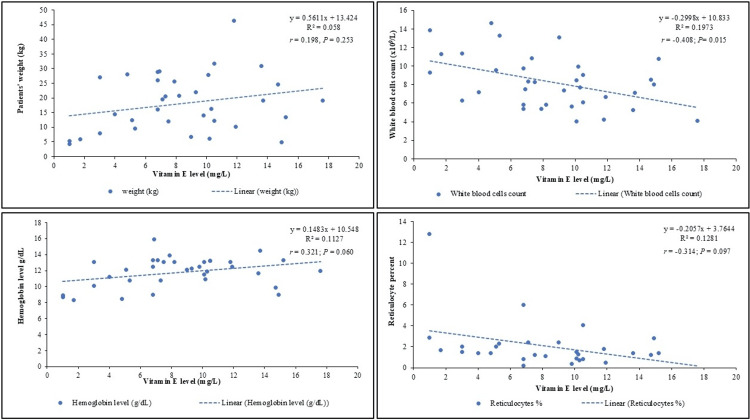
Correlation between vitamin E levels and patient’s weight, white blood cell count, hemoglobin levels, and reticulocyte percentage in children with cystic fibrosis Image credits: Hasan M. Isa, Fatema A. Alkharsi, Zahraa S. Mohamed, Zahra H. Isa, Batool H. Isa, Mahmood J. Ali, Afaf M. Mohamed.

Spearman’s correlation analysis revealed a significant negative correlation between vitamin E levels and WBC count (r = -0.408; P = 0.015), as shown in Figure 3.

No significant correlation was found between vitamin E levels and patient weight at presentation (r = 0.198; P = 0.253), serum vitamin D level (r = 0.203; P = 0.250), fasting cholesterol level (r = 0.087; P = 0.618), hemoglobin level (r = 0.321; P = 0.060), reticulocyte percentage (r = -0.314; P = 0.097), number of hospital admissions (r = -0.011; P = 0.950), and number of pulmonary exacerbations (r = 0.013; P = 0.943).

Upon comparison between vitamin E-deficient patients and those with normal vitamin E levels in terms of laboratory results, vitamin E-deficient patients had lower hemoglobin levels (P = 0.001), a higher reticulocyte percentage (P = 0.034), and a higher WBC count (P = 0.001) (Table 4).

**Table 4:** Laboratory results of patients with cystic fibrosis and vitamin E deficiency and those with normal levels Data are presented as number (%), mean ± standard deviation, or median (interquartile range). ^a^Student t-test; ^b^Mann-Whitney U test P-value < 0.05 is considered statistically significant. Bold values indicate statistical significance CI: confidence interval

Variables	Normal range	Vitamin E Level		P-value
		Low, n = 9 (25.7)	Normal, n = 26 (74.3)	
White blood cell count (x10^9^/L)	3.6-9.6	10.8 ± 2.9	7.4 ± 2.3	0.001^a^ (CI: -5.3, -1.4)
Hemoglobin level (g/dL)	12-14.5	10.2 ± 1.7	12.4 ± 1.6	0.001^a ^(CI: 0.9, 3.4)
Platelets count (x10^9^/L)	150-400	495 (367-583)	379 (289-444)	0.140^b^
Reticulocyte (%)	0.5-1.5	2 (1.5-2.3)	1.3 (0.8-2.1)	0.034^b^
Vitamin D level (nmol/L)	>50	29 (21-39)	33 (27-44)	0.465^b^
Cholesterol level (mg/dL)	139-200	105.9 ± 25.0	121.9 ± 27.9	0.139^a^ (CI: 37.5, 5.5)
Total protein (g/L)	64-82	71.2 ± 12.9	73.5 ± 9.6	0.592^a^ (CI: -10.7, 6.2)
Albumin (g/L)	35-52	43 (37-44)	44 (41-46)	0.328^b^
Globulin (g/L)	15-30	28 (22-38)	29 (25-34)	0.921^b^
Bilirubin (µmol/L)	5-21	5 (4-12)	5 (5-8)	0.651^b^
Alkaline phosphatase (µ/L)	50-136	34 (30-35)	22 (19-32)	0.130^b^
Alanine aminotransferase (µ/L)	<41	22 (18-58)	23 (19-42)	0.908^b^
Gamma-glutamyl transferase (µ/L)	5-55	18 (14-80)	12 (9-22)	0.102^b^

To identify predictors of vitamin E deficiency, a binary logistic regression was performed including the significant variables, which showed that none of these variables were considered independent risk factors of vitamin E deficiency (Table 5).

**Table 5:** Binary logistic regression of vitamin E deficiency selected risk factors in children with cystic fibrosis CI: confidence interval

Variable	Adjusted odds ratio	95% CI	P-value
Weight at presentation	0.960	0.834, 1.106	0.573
Bronchodilator (salbutamol) use	10.187	0.721, 144.000	0.086
White blood cell count	1.207	0.731, 1.992	0.462
Hemoglobin level	0.618	0.258, 1.480	0.280
Reticulocyte %	0.954	0.589, 1.546	0.850

On follow-up, six patients died during or after the study period with a mortality rate of 17.1%. Vitamin E-deficient patients had a higher mortality rate (n = 2/9, 22.2%) compared to those with normal levels (n = 4/26, 15.4%), but this difference was not statistically significant (P = 0.635).

## Discussion

This study showed a high prevalence of vitamin E deficiency among patients with CF in Bahrain (25.7%). Comparably, in Australia, the mean prevalence of vitamin E deficiency was 17.92%, which fell from 20.22% in 2007 to 13.89% in 2010 [6]. This reduction is due to increased awareness of the problem among this group of patients [6]. However, vitamin E deficiency is an infrequent condition, and it is mainly caused by irregularities in fat absorption or metabolism rather than low dietary intake [23]. Patients with CF had low serum levels of vitamin E due to fat malabsorption despite enzyme replacement therapy [9]. Pancreatic insufficiency, variable degrees of bile salt disturbance, and hepatobiliary dysfunction make fat-soluble vitamin deficiency frequent in patients with CF [3]. Moreover, chronic inflammation and respiratory exacerbations lead to increased consumption of these vitamins [9].

Vitamin E-deficient patients had significantly lower weight at presentation (P = 0.045) than those with normal levels but no difference in the BMI. This finding was expected as patients with weight faltering may also have poor adherence to pancreatic enzyme replacement therapy intake, fat, and fat-soluble vitamin supplementation [24]. Chronic fat malabsorption and increased metabolic expenditures due to lung disease can cause failure to gain weight and thrive [24]. However, the Sagel et al. [25] study found no significant differences in weight or BMI between patients with CF treated with antioxidants and those in the control group.

In this study, vitamin E-deficient patients had significantly higher WBC counts (P = 0.001) than those with normal levels. A high WBC count (leukocytosis) might indicate an obvious or hidden infection or inflammation in the body, as it reflects the normal response of the bone marrow to an infectious or inflammatory process [26]. Patients with CF are predisposed to both complications due to recurrent chest infections [22,27]. Moreover, patients with vitamin E deficiency are more prone to inflammation, infection, severe sepsis, and potentially septic shock [22,27]. Dang et al. [27] reported that the incidence of vitamin E deficiency was 30.2% in the infection group and 61.9% in the septic shock subgroup. Vitamin E is an important anti-inflammatory agent, and it has a marked effect in infectious diseases where immune phagocytosis is involved [5]. It is essential for stimulating the body's defense mechanisms, enhancing both humoral and cellular immune responses, and boosting phagocytic activity [5].

In the current study, vitamin E-deficient patients had significantly lower hemoglobin levels and higher reticulocyte percentages than the vitamin E-sufficient patients. This combination of low hemoglobin and reticulocytosis indicates a hemolytic type of anemia [28]. In line with our findings, Wilfond et al. [29] reported severe hemolytic anemia in three infants with CF that was caused by vitamin E deficiency. Hemolytic anemia is known to result from vitamin E deficiency; this finding has been extensively documented in the literature [22,30]. In vitamin E deficiency, hemolytic anemia is caused by reduced erythrocyte life span due to increased vulnerability to free-radical-induced erythrocyte membrane lysis [30]. Cellular oxidative stress is boosted by a change in the normal balance between the quantity and the function of the pro-oxidants and antioxidants [31]. It is known to produce reactive oxygen species (ROS), which are highly reactive free radicals [31]. ROS could rapidly interact with cellular macromolecules like membrane lipids, proteins, polysaccharides, and nucleic acids, leading to cell damage [31]. This damage may lead to premature hemolysis due to increased fragility of the red cell membrane [31]. Oral vitamin E supplementation can quickly correct in vitro hemolysis and enhance in vivo hematologic indices [29].

Although it was not statistically significant, vitamin E-deficient patients in our study had a higher median number of pulmonary exacerbations and hospital admissions. This finding was supported by the Hakim et al. [9] study, which reported that reduced serum levels of vitamin E were associated with an increased rate of pulmonary exacerbations in patients with CF even if the level was within the normal range. During an acute pulmonary exacerbation, serum levels of vitamin E are likely to be decreased due to higher consumption of antioxidant vitamins [9]. Moreover, Sagel et al. [25] reported that patients with CF treated with antioxidants had a significantly lower risk of first pulmonary exacerbation requiring antibiotics than the control group. On the contrary, Woestenenk et al. [10] found that higher serum alpha-tocopherol levels had no protective effect on pulmonary function in children with CF. Sagel et al. [25] also reported no significant difference in the hospitalization event rate between the antioxidant-treated and the control group. However, after 12 weeks of vitamin E supplements, Sagel et al. [25] reported modest improvements in weight and pulmonary functions in patients with CF.

High mortality in CF can be the result of disease severity either genotypically or phenotypically [32]. Patients with pancreatic insufficiency, delayed diagnosis, inadequate or inappropriate therapy, and those with recurrent pulmonary exacerbations are prone to poor outcomes [17]. In this study, six (17.1%) patients died on follow-up; two of them were vitamin E-deficient. Although not statistically significant, vitamin E-deficient patients had a higher mortality rate (33.3%) in comparison with vitamin E-sufficient patients (13.8%). Similarly, Dang et al. [27] reported that vitamin E-deficient critically ill patients had significantly higher mortality than patients without vitamin E deficiency.

Although supplementation of patients with CF with fat-soluble vitamins, including vitamin E, is a routine of care for all patients, this does not guarantee that the provided amount of vitamin E will be sufficient to treat vitamin E deficiency. Each 1 ml of fat-soluble vitamin supplementation in aqueous form (for example, AquaADEKs) provides 1743 mcg of vitamin A, 400 units of vitamin D, 48 mg of vitamin E, and 0.4 mg of vitamin K [33]. However, these amounts are very low as patients with CF require a higher amount of water-soluble vitamin E with the recommended amount of 400 mg daily for life due to their inability to secrete bile [34].

Vitamin D deficiency is another common problem in children with CF due to impaired absorption of fat-soluble vitamins, limited sunlight exposure, and low intake of vitamin D-containing foods or supplements [35]. Yet, in the present study, vitamin D deficiency was found in 82.4% of patients with CF. This figure is lower than that reported in healthy children from Bahrain, where vitamin D deficiency was found in 93.4% [36]. This difference can be explained by the fact that most patients with CF in our study were previously taking fat-soluble vitamins (ADEK), which contain the minimal recommended daily dose of vitamin D (400 IU) [33]. Nonetheless, the percentage of vitamin D deficiency in our study is much higher than that reported in Canada, where vitamin D deficiency was reported in 26% and 23% of children with CF in 2010 and 2011, respectively [37]. Moreover, in Australia, the prevalence of vitamin D deficiency fell from 22.11% in 2007 to 15.54% in 2010, with a mean prevalence of 16.96% [6]. Geographical location and climate changes can play an important role in determining vitamin D status due to the influence of ultraviolet B rays on the cutaneous production of pre-vitamin D3 [38].

Lipid digestion and absorption are usually impaired in patients with CF, as shown by Woestenenk et al. [39], who reported low concentrations of cholesterol, high-density lipoprotein cholesterol, and low-density lipoprotein cholesterol among their patients with CF. Similarly, most patients in the present study had low fasting cholesterol levels (82.9%). Gelzo et al. [40] also found that plasma cholesterol levels were significantly lower in patients with CF when compared to the control subjects. In patients with CF, despite impaired intestinal absorption of exogenous sterols that can stimulate the endogenous synthesis of cholesterol, the total serum cholesterol remains low. This could be attributed to CFTR dysfunction, which reduces cholesterol excretion from the blood, leading to its accumulation in liver cells and other tissues [40].

The current study was limited by the small sample size and being conducted at a single tertiary center. Accordingly, this study might not reflect the true prevalence of vitamin E deficiency among children with CF in Bahrain. However, since most children with CF in Bahrain are referred to our hospital, the chance of missing cases is minimal. Moreover, there are still some concerns about biases resulting from selective patterns of referrals, which may result in the underreporting of these patients. Another limitation is that the study relied on a vitamin E test used for clinical purposes, which has not been cleared or approved by the US Food and Drug Administration (FDA). Using a non-FDA-approved test might compromise the study's integrity and applicability, impacting both its scientific contribution and its practical implications. However, this test was the only available option in our country and was previously used by other studies [6,9,27]. Moreover, studies were scarce regarding vitamin E deficiency in patients with CF for comparison.

Despite these limitations, this study is a prospective analytical study with strict inclusion criteria. The findings of this study are important as it is the first study from our region about vitamin E deficiency among patients with CF that focuses on the correlation between vitamin E levels and pulmonary exacerbation, cholesterol, vitamin D, and other laboratory results. This type of study is essential for any pediatrician or gastroenterologist, as it can help them improve management plans as well as form a strong foundation for any future research.

## Conclusions

Vitamin E deficiency is a common problem among patients with CF in Bahrain. It is an important risk factor for morbidities. This study revealed that patients with vitamin E deficiency had lower weight at presentation, lower hemoglobin levels, a higher reticulocyte percentage, a higher WBC count, and lower salbutamol use than those with normal levels. However, there was no distinction in the number of previous admissions, pulmonary exacerbations, vitamin D deficiency, or low cholesterol levels between the two groups. In patients with CF, management of vitamin E deficiency should be hastened to avoid irreversible complications. Further studies that include a larger number of patients are needed to assess the effect of vitamin E supplementation in preventing pulmonary exacerbations in patients with CF and to determine the optimal dose of vitamin E required to achieve “maximum” clinical effectiveness.
